# Life-threatening small-bowel diverticular bleed treated by double-balloon enteroscopy in a patient refusing transfusion

**DOI:** 10.1055/a-2224-7759

**Published:** 2024-01-23

**Authors:** Laura A. Lucaciu, Alexandros Skamnelos, Alberto Murino, Nikolaos Lazaridis, Rocio Chacchi Cahuin, Regina Raymond, Edward J. Despott

**Affiliations:** 1 4965 Royal Free Unit for Endoscopy, Royal Free London NHS Foundation Trust, London, United Kingdom of Great Britain and Northern Ireland; 2 171090 University College London Institute for Liver and Digestive Health, London, United Kingdom of Great Britain and Northern Ireland

Double-balloon enteroscopy (DBE) is the gold standard for minimally invasive investigation and management of a vast array of small-bowel pathology 1 . Small-bowel diverticulosis is rare, with a prevalence of 1%–2% in the general population 2 . Complications including diverticular bleeding, perforation, and obstruction may occur in 10%–30% of cases 2 3 . We present a case of massive jejunal diverticular bleed successfully managed by DBE-facilitated endotherapy.

A 65-year-old man presented to our tertiary center with a short history of melena, abdominal pain, and symptomatic anemia, including dizziness and syncopal episodes at rest, on a background of severe COVID-19 infection. He was transferred to the intensive care unit (ICU) as his hemoglobin dipped to a critically low level (32g/L) during admission. Despite counseling regarding risk, the patient refused blood transfusion due to religious beliefs.

Emergent esophagogastroduodenoscopy and colonoscopy were unrevealing. Given ongoing bleeding, two separate computed tomography angiograms of the abdomen–pelvis were subsequently performed, but failed to show the bleeding source. Small-bowel capsule endoscopy showed multiple diverticula in the jejunum with blood content in the small bowel and colon. Emergency anterograde DBE was performed under general anesthesia in the ICU, showing severe jejunal diverticulosis with large vessels in the diverticula sacs. Active bleeding was identified at a sizable small-bowel diverticulum, and three endoclips were deployed for successful hemostasis ( Video 1 ). The hemoglobin returned to acceptable levels during admission following treatment with intravenous iron infusions, tranexamic acid, and erythropoietin. During 3 years of follow-up, there was no recurrence of bleeding.

Hemostasis of jejunal diverticular bleeding achieved by double-balloon enteroscopy-facilitated endotherapy.Video 1

This case demonstrates that DBE-facilitated endotherapy is a safe and effective approach in the context of life-threatening jejunal diverticular bleeding, even in a challenging scenario with a critically low hemoglobin in a patient refusing transfusion.

Endoscopy_UCTN_Code_CCL_1AC_2AF

## Notes

Endoscopy E-Videos https://eref.thieme.de/e-videos
*E-Videos* is an open access online section of the journal *Endoscopy* , reporting on interesting cases and new techniques in gastroenterological endoscopy. All papers include a high-quality video and are published with a Creative Commons CC-BY license. Endoscopy E-Videos qualify for HINARI discounts and waivers and eligibility is automatically checked during the submission process. We grant 100% waivers to articles whose corresponding authors are based in Group A countries and 50% waivers to those who are based in Group B countries as classified by Research4Life (see: https://www.research4life.org/access/eligibility/ ). This section has its own submission website at https://mc.manuscriptcentral.com/e-videos .
